# The Polybrominated Diphenyl Ether Mixture DE-71 Is Mildly Estrogenic

**DOI:** 10.1289/ehp.10643

**Published:** 2008-01-29

**Authors:** Minerva Mercado-Feliciano, Robert M. Bigsby

**Affiliations:** 1 Department of Pharmacology and Toxicology and; 2 Department of Obstetrics and Gynecology, Indiana University School of Medicine, Indianapolis, Indiana, USA

**Keywords:** CYP1A, CYP2B, DE-71, endocrine disruptors, estrogens, MCF-7, mice, ovariectomized, PBDEs, polybrominated diphenyl ethers

## Abstract

**Background:**

Polybrominated diphenyl ethers (PBDEs) are widely found in the environment, and they may act as endocrine disruptors.

**Objective:**

Our goal in this study was to test the PBDE mixture DE-71 for estrogenic activity.

**Methods:**

We used proliferation of cultured breast cancer cells (MCF-7) and trophic effects in the reproductive tracts of ovariectomized mice as estrogen bioassays. DE-71 was administered to mice by subcutaneous injection (sc) or oral gavage (po), alone or in combination with estradiol, for 3 or 34 days. Liver weights and cytochrome P450 enzyme activities were also measured.

**Results:**

DE-71 increased MCF-7 cell proliferation, and this was prevented by antiestrogen. DE-71 cotreatment reduced the effect of estradiol in MCF-7 cells. In the mouse 3-day assay, DE-71 administered alone had no effect on uterine weight, uterine epithelial height (UEH), or vaginal epithelial thickness (VET); however, when DE-71 was administered as a cotreatment, it potentiated estradiol’s effect on uterine weight. DE-71 administered sc to BALB/c mice for 34 days slightly increased UEH and VET, and attenuated the estradiol-induced increase in UEH; these effects were not seen in BALB/c mice treated po or in C57BL/6 mice treated sc. DE-71 increased liver weight in BALB/c, C57BL/6, and estrogen receptor-α knockout mice. We also found an increase in liver cytochrome P450 1A (CYP1A) and CYP2B activities when DE-71 was administered po, but only CYP2B increased after sc treatment.

**Conclusion:**

DE-71 behaves as a weak estrogen. In mice, the treatment route and duration determined if DE-71 was estrogenic. BALB/c mice are more susceptible to DE-71 effects in estrogen target tissues than C57BL/6 mice. DE-71 increased liver weight independently of estrogen receptor-α.

The polybrominated diphenyl ethers (PBDEs) are a series of 209 possible brominated diphenyl ethers (BDEs) that differ in the number and position of bromine atoms [Agency for Toxic Substances and Disease Registry (ATSDR) 2004]. Three kinds of PBDE blends were manufactured and marketed as standard mixtures: Penta-BDE (containing penta-, tetra-, and some hexabrominated congeners), Octa-BDE (containing from hexa- to decabrominated congeners), and Deca-BDE (97% deca-BDE and 3% nonbrominated congeners). Standard mixtures of PBDEs have been used extensively as flame retardants over the past 30 years in a variety of consumer products (e.g., plastics, foams, electronics). The PBDEs are very stable compounds and are not chemically bonded to material they are intended to protect from burning. Therefore, it is not unexpected that PBDEs are being found more and more in environmental media [reviewed by Domingo (2004), Hites (2004), and Law et al. (2006)], and possible exposure to them has become a public health concern.

The endocrine-disrupting potential of PBDEs has been studied, mostly in regard to their effect on thyroid hormone homeostasis. Commercial PBDE formulations reduce thyroid hormone levels and induce thyroid hyperplasia in rodents (Darnerud et al. 2007; Stoker et al. 2004). Increased thyroid hormone clearance by induction of metabolic enzymes, and displacement of thyroxine from its transport protein have been suggested as mechanisms of thyroid disruption (reviewed by Siddiqi et al. 2003). Neurobehavioral alterations were observed in rodents treated neonataly with PBDEs (Viberg et al. 2002, 2003), but these alterations have not been linked to hormonal activity. Recent studies have linked PBDE to estrogenic effects *in vitro* (Meerts et al. 2001) and to adverse effects in sexual development and behavior in rodents (Ceccatelli et al. 2006; Kuriyama et al. 2005; Lilienthal et al. 2006; Stoker et al. 2004).

*In vivo* estrogen bioassays are classically based on responses of reproductive organ tissues in rodents. Estrogens induce many physiological changes in the mammalian female reproductive tract and mammary glands. Immature or ovariectomized (OVX) adult rodents have served as the standard bioassay model for estrogenic activity. In the adult mouse, estrogen target organ size and histologic characteristics regress to a nonstimulated state after ovariectomy, thus providing a model free of endogenous estrogens in which their physiologic effects can be studied. In OVX animals, estrogens increase uterine wet weight (Evans et al. 1941; Gordon et al. 1986) and uterine epithelial height (UEH) and vaginal epithelial thickness (VET) (Suzuki et al. 1996; Ulrich et al. 2000) after a few days of treatment. In the uterus, the columnar epithelial cells become taller, with a concomitant increase in cytoplasmic volume; they also proliferate, causing overcrowding and a pseudostratified appearance. In the vagina, the single squamous epithelial layer of the OVX mouse becomes a multicell layer after estrogen treatment.

Breast cancer cells in culture also serve as an estrogen bioassay. Although most normal mammary epithelial cells have no estrogen receptors (ERs) and depend on stromal interactions in their response to estrogen, carcinomas often express ERs, and estrogens induce their growth (Hahnel and Twaddle 1971; Osborne et al. 1985; Welsch et al. 1981). The fact that estrogens increase proliferation of neoplastic mammary epithelium *in vitro* is the basis of cell proliferation assays (Weichselbaum et al. 1978).

*In vivo* uterotrophic assays and *in vitro* breast cancer cell proliferation assays have been used extensively to assess the estrogenicity of environmental chemicals (Clode 2006; Soto et al. 1995). In the present study, we used an estrogen-responsive human cancer cell line, MCF-7, and the OVX mouse as bioassay models to assess the estrogenicity of DE-71, a standard Penta-BDE mixture of PBDEs commonly used in consumer goods.

## Materials and Methods

### Test chemicals

We purchased dimethyl sulfoxide (DMSO), 1,3,5[10]estratriene-3,17-β-diol [17β-estradiol, (E_2_)], and β-estradiol-3-benzoate (EB) from Sigma-Aldrich (St. Louis, MO). Corn oil was purchased from ICN Biomedicals Inc. (Aurora, OH). Fulvestrant [ICI 182 780 (ICI)] was a gift from Astra Zeneca (Macclesfield, Cheshire, UK). The PBDE congener mixture DE-71 (lot no. 9550OF05A) was a gift from the Great Lakes Chemical Corporation (West Lafayette, IN); the congener composition was reported previously (Qiu et al. 2007). There were no detectable dioxins in the DE-71 lot used, but it was not tested for furan content (Hites R, Qiu X, personal communication). DMSO was used as the primary solvent for all treatment chemicals, and the DMSO solutions were further diluted in corn oil for animal treatments. DE-71 remained in solution in DMSO at all doses used, but vials were still vigorously vortexed for several seconds immediately before treatment. DE-71 prepared for *in vivo* treatments was maintained in suspension in corn oil by continuous stirring while the needle was filled, and animals were dosed immediately.

### Cell proliferation measurements

To measure cell proliferation, we used the colorimetric assay developed by Mosmann (1983) with minor modifications. Cells were regularly maintained in growth medium [i.e. minimum essential media (MEM; Gibco/Invitrogen; Carlsbad, CA) supplemented with l-glutamine (2 mM; Gibco/Invitrogen), nonessential amino acids (0.1 mM; Gibco/Invitrogen), HEPES buffer (10 mM, Gibco/Invitrogen), and 5% vol/vol bovine growth serum (Hyclone, Logan, UT)]. To minimize basal hormonal activity during assays, cells were incubated in basal medium (i.e., phenol red free MEM supplemented with 2 mM l-glutamine, 0.1 mM nonessential amino acids, 10 mM HEPES, and 3% vol/vol dextran-coated charcoal-stripped bovine growth serum).

For the cell proliferation assay, cells cultured with growth medium were plated in 24-well dishes (15,000 cells/well). The day after plating, culture medium was changed to basal medium. Starting 2 days after changing to basal medium, cells were cultured in treatment medium (basal medium plus treatment) for a total of 10 days, changing the treatment medium every 2 days. On day 10, cells were incubated in tetrazolium MTT [3-(4,5-dimethylthiazolyl-2)2,5-diphenyltetrazolium bromide] for 2 hr, then lysed in acid iso-propanol. Reduced (blue) MTT absorbance was read at 570 nm.

To confirm the MTT absorbance correlated with cell growth and not increased metabolic activity, in some experiments we included additional plates treated in parallel for DNA determination. After 10 days of treatment, cells were washed twice with phosphate-buffered saline and incubated for 10 sec in cold methanol. After removing the methanol, we allowed cells to dry at room temperature. Cells were then dissolved in 0.5 M sodium hydroxide by rocking in a humidified 37°C chamber for 30 min. Samples were then transferred to microtubes and incubated at 65°C for 1 hr. Sample (100 μL) was diluted in TNE buffer (10 mM Tris, 0.1 M NaCl, 1 mM EDTA, pH 7.4) containing 0.1 mg/mL Hoescht 33258 dye (Polysciences, Warrington PA), and neutralized with an equimolar amount of hydrochloric acid. Fluorescence was measured in a Hoefer TKO 100 DNA Fluorometer (Hoefer Scientific Instruments, San Francisco, CA) against a salmon sperm DNA standard (Invitrogen, Carlsbad, CA).

### Animal treatments

All procedures performed on animals were approved by the Institutional Animal Care and Use Committee of the Indiana University School of Medicine. Animals were treated humanely and with regard for the alleviation of suffering. Adult BALB/c and wild-type (WT) C57BL/6 mice were purchased from Harlan (Indianapolis); ER-αKO mice (in C57BL/6 background for > 10 generations) were derived from an in-house colony. Animals were ovariectomized at 6–8 weeks of age, and 3 weeks later, they were treated for either 3 or 34 days with vehicle or test compound. In brief, groups of five or six animals were treated daily by either subcutaneous injection (sc) or oral gavage (po) with either vehicle control, EB (10 μg/kg), or DE-71 (50 mg/kg for 34 days or with 75, 150, or 300 mg/kg for 3 days). Some groups were cotreated with EB plus DE-71. Chemicals were first dissolved in DMSO and then diluted in corn oil and administered at 0.1 mL for po or at 10–20 μL for sc. Doses were prepared based on the average body weight measured for each group on the first day of treatment. On the day after the last treatment, animals were sacrificed by decapitation, and blood was collected by exsanguination. Serum was collected and stored at –20°C until analysis for individual BDE congeners and their hydroxylated metabolites [reported by Qiu et al. (2007)]. The liver was perfused in place with phosphate-buffered saline through the hepatic portal vein. The uterus and liver were weighed and expressed on a per gram of body weight basis. One uterine horn and the vagina were fixed in Bouin’s solution overnight. The liver was flash-frozen in liquid nitrogen and then stored at –70°C. The fixed uterus and vagina were embedded in paraffin, and 5-μm cross-sections were stained with hematoxylin and eosin for analysis by light microscopy. Using an image analysis program (IPLab, version 3.5 imaging software; Scanalytics Inc., Fairfax, VA), UEH and VET were measured as estrogen-sensitive end points.

### Cytochrome P450 (CYP) activity assays

For each animal, about 0.2 g of frozen liver was homogenized in 1 mL high-sucrose buffer [0.15 M potassium chloride, 0.5 M Tris, 1 mM EDTA, 0.25 M sucrose, 0.2 mM phenylmethylsulfonyl fluoride (PMSF), 20 μM butylated hydroxytoluene (BHT), pH 7.4] and centrifuged at 9,000 × *g* for 20 min at 4°C. To obtain the microsomal fraction, we centrifuged the resulting supernatant at 105,000 × *g* and 4°C for 60 min; the pellet was washed in potassium pyrophosphate buffer (0.1 M potassium pyrophosphate, 1 mM EDTA, 0.2 mM PMSF, 20 μM BHT, pH 7.4) by resuspending it using disposable homogenizing microtubes and pestles (Kontes Glass Company, Vineland, NJ). The protein content of the microsomal preparation was determined using the Pierce BCA Protein Assay Kit (Pierce, Rockford, IL) against a bovine serum albumin standard. The liver samples from each animal were assayed in duplicate.

We measured 7-ethoxyresorufin *O*-dealkylation (EROD; CYP1A) activity and 7-pentoxy-resorufin *O*-dealkylation (PROD; CYP2B) activity by mixing 5 μL of sample with 5 μL 250 μM NADPH and 3.4 μL 0.6 mM 7-ethoxyresorufin or 7-pentoxyresorufin in 1.2 mL 0.1 Tris buffer at 37°C. After allowing the mixture to equilibrate for 1 min, fluorescence was measured at 530 nm excitation and 585 nm emission in a fluorometer at 1-sec intervals over the course of 1 min. Similar measurements were made with six different resorufin concentrations to determine a standard curve. The slope of the linear range for each activity assay (Δfluorescence/sec) was converted to moles of resorufin per gram per second (mol/g*sec) using the standard curve and the protein content of each sample.

### Statistics

All statistics were performed using GraphPad Prism, version 3.0a, for Macintosh (GraphPad Software, San Diego, CA). For each statistical analysis, we used Bartlett’s test to determine if groups had unequal variances. Group averages with equal variances were compared to each other by either one-way analysis of variance (ANOVA) with Tukey post-test or unpaired *t*-test as appropriate. Group averages with unequal variances were compared to each other by *t*-test with Welch’s correction. Groups treated with DE-71 alone were analyzed against vehicle controls. Groups cotreated with DE-71 and EB were analyzed against controls treated with EB alone. All values are expressed as mean ± SE. Groups were considered statistically different if the result of ANOVA with Tukey post-test or *t*-test (two-tailed) had *p* < 0.05; however, in some instances, means were considered of borderline statistical significance if 0.05 < *p* < 0.10. DE-71 dose–response studies were also subjected to regression analysis using three curve fitting models: *a*) linear: response = (slope × dose) + intercept; *b*) sigmoidal: response = minimum + (maximum – minimum) ÷ {1 + 10^[Log(EC50) – Log(dose)]^ × Hillslope}; *c*) modified Gaussian distribution: response = minimum + ({maximum – minimum} × exp{–[log(dose) – *A*] ÷ slope}^2^, where *A* = Log(EC_50_) + 0.833 × slope, and the EC_50_ is the median effective concentration. The best fitting curve appears in graphs if *R*^2^ > 0.8.

## Results

### Cell proliferation assays

**Figure 1:**
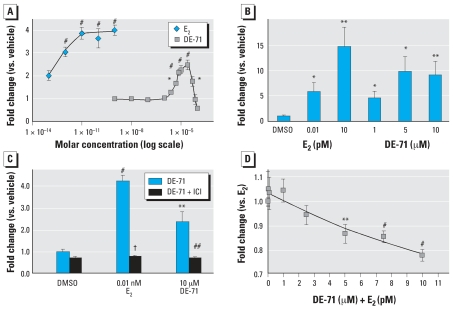
DE-71 induced proliferation of MCF-7 cells treated for 10 days with DE-71 and/or E_2_ at the indicated concentrations. Proliferation was measured using the MTT assay (*A, C, D*) or a DNA assay (*B*). The antiestrogen fulvestrant (ICI, 10 nm) blocked the effect of DE-71 (*C*). DE-71 blocked the effect of E_2_ in a dose-dependent manner (*D*). Values shown are mean ± SE of three to seven independent assays. *p < 0.05, **p < 0.01, and ^#^*p* < 0.001 vs. vehicle; ^##^*p* < 0.01, and ^†^*p* < 0.001 vs. treatment without ICI.

We compared the capacity of DE-71 to increase breast cancer cell (MCF-7) proliferation with that of E_2_. Both E_2_ and DE-71 were able to significantly increase cell number, as determined by the MTT assay (Figure 1A). DNA assays confirmed this finding, indicating that the increase in reduced MTT was caused by an increase in cell number and was not merely an effect on the cell redox systems (Figure 1B). The effects of both E_2_ and DE-71 were negated by cotreatment with the estrogen antagonist ICI (Figure 1C). DE-71 produced a biphasic dose–response curve, suggesting that it may have been toxic to MCF-7 cells at concentrations > 2.5 × 10^–5^ M (Figure 1A). At those same concentrations, we observed an accumulation of a white precipitate, suggesting a solubility problem. Cotreatment of cells with both E_2_ and DE-71 resulted in a lesser increase in cell proliferation compared with E_2_ alone, suggesting an antagonistic effect of DE-71 on E_2_-induced cell proliferation (Figure 1D). Antagonism was dose dependent; the highest dose tested corresponds to that which produced maximal proliferative effect when cells were treated with DE-71 alone.

### Three-day mouse estrogenic end points

**Figure 2:**
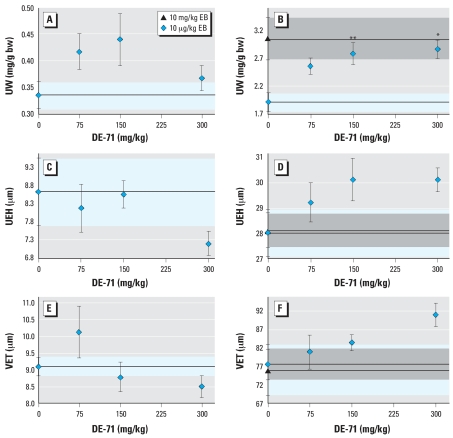
Dose effects of DE-71 on uterus and vagina in ovariectomized BALB/c mice treated sc for 3 days with DE-71 alone at the indicated doses (*A,C,E*) or with 10 μg/kg EB plus DE-71 (*B,D,F*). Abbreviations: bw, body weight; UW, uterine weight. (*A,B*) UW. (*C,D*) UEH. (*E,F*) VET. For (*A–F*), control means are shown as a dashed line; blue shaded areas indicate SE. Effects after 3 days of treatment with 10 mg/kg of EB are shown as a reference for maximal estrogen effect on uterine weight (*B*), UEH (*D*), and VET (*F*); dashed lines indicate the mean; gray shaded areas indicate SE. Values represent means ± SE of 8–10 mice per group. *p < 0.05, and **p < 0.01 indicate that individual UWs were significantly different from EB controls.

We used a 3-day treatment regimen in OVX BALB/c mice to assess DE-71 effects at three doses (75, 150, and 300 mg/kg); groups of animals were also treated with 10 μg/kg EB plus DE-71 at each of these doses. Uterine wet weight, UEH, and VET were used as estrogenic end points. DE-71 administration by sc alone had no statistically significant effect (Figure 2). However, the increase in uterine wet weight induced by 10 μg/kg EB was enhanced by DE-71 in a dose-dependent manner; this enhanced response was equivalent to the maximal estrogen effect produced by 10 mg/kg EB (Figure 2B). Oral DE-71 administration had no effect on any of the estrogenic parameters measured (data not shown).

Subcutaneous DE-71 treatment alone had no effect on UEH or VET (Figure 2C, 2E). When mice were cotreated with DE-71 and EB, there was an increasing dose–response trend for UEH and VET (Figure 2D, 2F). However, only borderline statistical significance was achieved for UEH in the highest dose group, and neither trend yielded a good fit by regression analysis.

### Thirty-four-day mouse estrogenic end points

We determined estrogenic effects in ovariectomized BALB/c and C57BL/6 WT and ER-αKO mice treated long-term (34 days). We examined uterine and vaginal parameters as in the 3-day assay. As with the 3-day assay, oral DE-71 administration had no effect on any of the estrogenic parameters measured in BALB/c mice (data not shown); C57Bl/6 mice were not treated orally.

**Figure 3:**
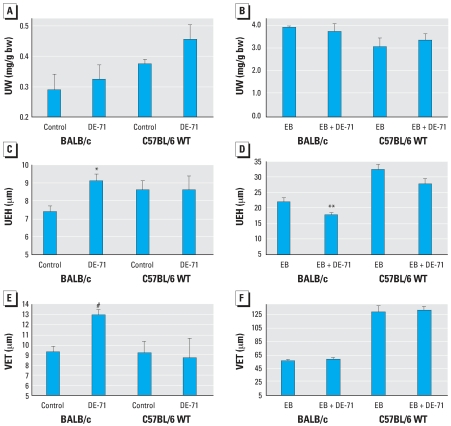
Effects of DE-71 in uterus and vagina of ovariectomized BALB/c and C57BL/6 WT mice treated for 34 days with DE-71 alone (*A,C,E*) or E_2_ plus DE-71 (*B,D,E*). Abbreviations: bw, body weight; UW, uterine weight. (*A,B*) Relative UW. (*C, D*) UEH. (*E,F*) VET. Values shown are mean ± SE of 5–10 mice per group. *p < 0.05, **p < 0.01, and ^#^*p* < 0.001 vs. corresponding control.

Treatment for 34 days with sc DE-71 alone produced no significant change in uterine weight (Figure 3A). There was a large increase (8- to 12-fold; *p* < 0.001) in uterine weight of WT mice after E_2_ treatment. The uterine weight response to 10 μg/kg EB at 34 days was similar to the increase produced by 10 mg/kg EB for 3 days; that is, the 34-day EB treatment produced the maximal uterotrophic effect. EB-induced uterine weight was unaffected by cotreatment with DE-71 (Figure 3A).

In BALB/c mice, DE-71 administered sc for 34 days caused a 23% increase in UEH (Figure 3C) and a 33% increase in VET (Figure 3E). When administered alone, DE-71 had no effect on these parameters in C57BL/6 mice. In contrast, we observed a small but statistically significant decrease in the estrogen-induced UEH increase in BALB/c mice cotreated with sc DE-71 (Figure 3D). The similar decrease in UEH in cotreated C57BL/6 mice was not statistically significant. Cotreatment did not alter the EB-induced increase in VET (Figure 3F).

As expected, EB treatment had no effect on ER-αKO uterine weights, UEH, or VET, nor did DE-71 have an effect on any of the uterine or vaginal parameters in either WT C56BL/6 mice (Figure 3) or ER-αKO animals (data not shown).

### Liver end points

**Figure 4:**
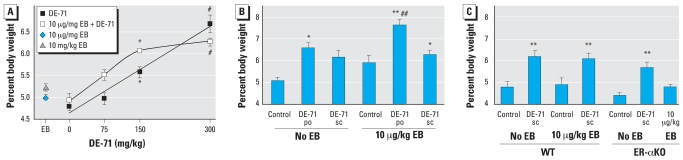
Effects of DE-71 on liver weight of mice were treated for either 3 (*A*) or 34 (*B,C*) days with DE-71 alone or with E_2_ and DE-71. Controls for the 34-day time point were treated po with vehicle. Values shown are mean ± SE (*n* = 5–10 mice per sc group; *n* = 4–5 mice per po group). *p < 0.05, **p < 0.01, and ^#^*p* < 0.001 vs. vehicle. ^##^*p* < 0.05 vs. DE-71 at the same dose without EB.

We determined liver weights and activities of CYP1A and CYP2B enzymes. We found a 20–51% increase in liver weight in BALB/c mice treated with DE-71 for 34 days compared with vehicle control, whereas livers of 3-day treated mice increased in weight up to 39% in a dose-dependent manner (Figure 4).

EB treatment did not increase liver weight in BALB/c mice. However, EB potentiated the effect of po DE-71 but had no effect on sc DE-71 treatments (Figure 4B). As with BALB/c mice, the livers of DE-71 treated C57BL/6 WT mice were 27–29% larger than vehicle-treated controls. E_2_ administered alone or in combination with DE-71 had no effect on liver weights of C57BL/6 WT mice. The size of ER-αKO mouse livers increased 30% after DE-71 treatment (Figure 4C).

**Figure 5:**
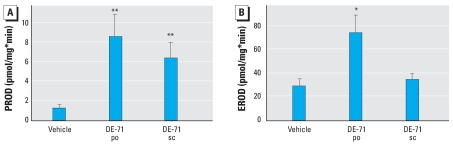
Effects of DE-71 on liver microsomal CYP activity in BALB/c mice treated for 34 days with DE-71 alone or with EB plus DE-71. (*A*) PROD (CYP2B) activity. (*B*) EROD (CYP1A) activity. Values shown are mean ± SE. *n* = 8 mice per group. *p < 0.05, and **p < 0.01 vs. vehicle.

We found a large increase in PROD activity in DE-71–treated BALB/c mice compared with controls, about 7-fold in the po-treated group and 5-fold in the sc-treated group (Figure 5A). Still, PROD activity in the DE-71–induced animals was much lower than EROD activity in vehicle-treated animals (i.e., the maximal PROD was about one-third the minimal EROD activity). Liver microsomal EROD activity also increased (2.5-fold), but only for po-treated mice (Figure 5B). EB treatment had no effect on either EROD or PROD activity (data not shown).

## Discussion

DE-71 exhibited both estrogenic and anti-estrogenic effects in the MCF-7 cell proliferation assay and in the adult ovariectomized mouse model, a behavior expected from a weak ER agonist (reviewed by Lerner and Jordan 1990). The observation that ICI prevented DE-71 from increasing cell number in the MCF-7 bioassay suggests the involvement of an ER.

To date, this is the first report of a study in which the estrogenic action of a PBDE mixture has been examined using standard rodent bioassay end points. The magnitude of the effects seen in our *in vivo* studies was similar to those produced by treatment of ovariectomized rats with 200 mg/kg BDE-47, a major component of DE-71 (Dang et al. 2007). The type of response observed (agonist or antagonist) was dependent upon the duration of exposure to DE-71. In the 3-day assay DE-71 administered alone produced no estrogenic effects; however, when it was administered for 34 days, it produced hypertrophy of the uterine epithelium and hyperplasia of the vaginal epithelium. When administered with EB in the 3-day treatment schedule, DE-71 enhanced the estrogen effect, whereas in the 34-day treatment schedule DE-71 produced small anti-estrogenic effects. Thus, pharmacokinetic considerations are paramount when designing further studies of PBDE action *in vivo*.

We used the ER-αKO mouse to determine if *in vivo* effects of DE-71 were mediated by ER-α; however, because there was a lack of any estrogenic effects of DE-71 in C57BL/6 WT mice, this experiment was not informative. The observation that DE-71 could enhance estrogenic effects beyond those produced by a saturating dose of EB suggests that PBDEs modify estrogen action through nonclassical pathways, as has been proposed for other xenobiotics such as β-hexachlorocyclohexane (Hatakeyama et al. 2002; Steinmetz et al. 1996).

PBDEs are suspected to behave as estrogens because of the similarity of their chemical structure and properties to other xenobiotics, mainly the polychlorinated biphenyls [reviewed by Hooper and McDonald (2000), Meerts et al. (2001), and Pijnenburg et al. (1995)]. Furthermore, hydroxylated metabolites of PCBs have been shown to exert estrogenic effects [reviewed by Bigsby et al. (2005) and Blair et al. (2000)]; therefore, it may be reasonable to expect that hydroxylated forms of PBDEs would also be estrogenic. Several researchers have shown that individual BDE congeners or certain synthetic hydroxylated congeners could exert estrogenic effects in cultured cells. In estrogen-responsive transcription reporter assays, BDE-28 and BDE-100, as well as the 4′-hydroxy forms of BDE-30 and BDE-119, proved to be estrogenic (Meerts et al. 2001). In addition, several BDE congeners found in DE-71 were mildly anti-estrogenic in the same assay. Likewise, Hamers et. al. (2006) observed weak estrogenic activity by several low-brominated BDEs, weak anti-estrogenic activity for tetra- and heptabrominated BDEs and 6OH-BDE-47, and neither activity for the DE-71 mixture.

Our results show that in the OVX mouse sc DE-71 produced very small estrogenic effects, whereas po DE-71 had no effect. Because DE-71 had estrogenic effects in cell culture, the lack of effect in po-treated mice suggests rapid clearance via liver metabolism. However, when we analyzed plasma of mice that had been treated with DE-71 for 34 days, we found similar amounts of parent compounds and metabolites in the blood of either sc- or po-treated animals, with the exception of BDE-153, which was 5 times higher in the po group (Qiu et al. 2007). Furthermore, the concentration of total DE-71 congeners was approximately 1,000–2,000 ng/mL, similar to what would be achieved with 2–4 μM treatment in culture, where 1 μM was minimally effective and 5 μM showed an effect that was approximately 50% of the maximal E_2_-induced effect. Staskal et al. (2005) found that non-metabolized BDE-47 is rapidly cleared in the mouse. This process is mediated by urinary proteins and may be saturated after repeated exposure (Diliberto et al. 2007; Staskal et al. 2006). Because the animals were treated long-term with a high dose of DE-71, it is possible that the concentrations we found in blood represent a steady-state level reached when urinary clearance is at its maximum capacity. Although this may explain why the po- and sc-treated animals had similar blood concentrations of most PBDEs, it does not explain why the sc route produced estrogenic effects while the po route did not. Another explanation is that more PBDE congeners may reach estrogen targets when administered sc rather than po. It is possible that sc-administered PBDEs reached higher levels in peripheral tissue than liver compared with po-administered PBDEs by avoiding the high activity of conjugating (phase II) enzymes in the liver and gut (Cassidy and Houston 1984; Li et al. 2004). It is also possible that PBDEs are activated by CYP in peripheral tissues such as adipose or at the estrogen-target tissues (Shimada et al. 2003, Yoshinari et al. 2006). Others have also seen higher efficacy of sc dosing over po dosing of estrogens, as is the case of the xenoestrogen bisphenol A (Berger et al. 2007) and steroidal estrogen (Savvas et al. 1992).

The MCF-7 cell proliferation assay used here to test DE-71 is probably more sensitive than the reporter gene expression system used by others (Hamers et al. 2006; Legler et al. 1999) due to the longer time of incubation with the chemical (10 days vs. 24 hr), thereby allowing accumulation of both the PBDE congeners inside the cell [increased intracellular concentration (Mundy et al. 2004)] and the estrogenic effect (cell growth). Alternatively, MCF-7 cells are known to express CYP enzymes (Barber et al. 2006; Peters et al. 2004), and it may be that during the 10-day incubation they metabolically convert BDE congeners to more active hydroxylated forms.

Other researchers have shown that both BDE-99 and DE-71 interfere with rodent sexual development after prenatal exposure (Ceccatelli et al. 2006; Kuriyama et al. 2005; Lilienthal et al. 2006), but specific hormonal activity involved was not demonstrated. As noted by Ceccatelli et al. (2006), PBDE remains in the offspring for months after birth, making it impossible to determine if the observed increase in expression of estrogen target genes during adulthood was due to developmental defects or adult hormone-like effects (or a combination of both). Although such research is suitable to assess the sensitivity to developmental effects, it does not define these effects as estrogenic. The pubertal development protocol used by Stoker et al. (2004) and the adult gonadectomized rodent model used here are more suitable to assess estrogenic activity by looking at well-known responses to estrogen after chemical challenge. Classic estrogenic responses such as increased uterine weight, UEH, and VET in the adult OVX mouse are a strong indication of the involvement of estrogen-signaling pathways and are standard methods to assess estrogenicity of a chemical (Clode et al. 2006).

We found differences in responses between mouse strains: The uterine and vaginal epithelium seems to be more sensitive to the effects of DE-71 in BALB/c mice than in C57BL/6 mice. Others have shown that C57BL/6 mice exhibit higher sensitivity to estrogen compared with other strains (Silberberg and Silberberg 1951; Spearow et al. 1999, 2001), but BALB/c mice were not included in those comparisons. Although our findings may illustrate a real difference in estrogen sensitivity between the BALB/c and C57BL/6 strains, an alternative explanation is that between-assay variability makes it difficult to measure small estrogenic effects (Ashby et al. 2004; Thigpen et al. 2002; Tinwell et al. 2000).

DE-71 increased liver weight in both BALB/c and C57BL/6 mice treated for 34 days, an effect shown previously in rats (Stoker et al. 2004; Zhou et al. 2001, 2002) and mink (Martin et al. 2007). The fact that a similar increase in liver weight occurred in ER-αKO mice indicates that this is not dependent on ER-α signaling. An increase in liver weight may occur in parallel with histologic changes such as hepatocytomegaly, acidophilic cytoplasm, binucleated hepatocytes, and eosinophilic bodies (Hardy et al. 2002; Son et al. 2007). Although we did not analyze the tissue histologically, others have shown that oral administration of DE-71 to rats for up to 90 days resulted in increased liver weights, with concomitant histologic changes (International Programme on Chemical Safety 1994). In BALB/c mice treated for 3 days, DE-71 increased liver weight in a dose-dependent manner only when administered po. Interestingly, EB potentiated the DE-71–induced increase in liver weight in BALB/c but not in C57BL/6 mice. Previous studies have shown that an estrogen-induced increase in liver weight in mice is strain dependent (Buchanan et al. 2002; Duffel et al. 1981; Goodin et al. 2002; Wang et al. 2004).

Because DE-71 treatment increased liver weight while at the same time diminishing the action of administered EB on the uterine epithelium (34-day treated mice only), it is possible that DE-71 can alter liver metabolic pathways that regulate systemic estrogen activity. A major pathway responsible for E_2_ deactivation is catalyzed by CYP enzymes, and of those CYP1A have the highest activity for E_2_ hydroxylation (Lee et al. 2003). A less active enzyme, CYP2B, also may be involved in E_2_ hydroxylation (Acevedo et al. 2005). Induction of hepatic EROD (CYP1A activity) and PROD (CYP2B activity) has been shown in rats (Stoker et al. 2004; Zhou et al. 2001, 2002) and mink (Martin et al. 2007) treated with DE-71 and mice treated with Bromkal 70-5 DE, a PBDE mixture with congener composition similar to DE-71 (Lundgren et al. 2007). In the present study, EROD (CYP1A) was increased by DE-71 but only when administered orally. PROD (CYP2B) was increased by DE-71 regardless of the route of administration, but the increase occurred to a much greater extent when the po route was used. Because the small antiestrogenic effect observed in the uterine epithelium occurred only when DE-71 was administered sc, it does not appear that this effect can be attributed to altered estrogen metabolism through increased CYP1A or CYP2B.

Individual PBDE congeners are known to induce expression of CYP2B, but the induction of CYP1A activity by PBDEs is most likely due to contamination with polybrominated dibenzo-*p*-furans (Hanari et al. 2006; Kuiper et al. 2006), which are known to induce aryl hydrocarbon receptor signaling in mammals (Olsman et al. 2007). Sanders et al. (2005) found that both DE-71 and its three main component congeners (BDEs 47, 99, and 153) increased *CYP2B* gene expression in rats, but that only DE-71 and not the individual congeners strongly up-regulated CYP1A, thus suggesting that the CYP1A increase was due to furan contamination of the DE-71 mixture. Because furans do not induce CYP2B, PROD induction is most likely mediated by PBDE activation of the constitutive androstane receptor, CAR (reviewed by Yamada et al. 2006).

In summary, the PBDE mixture DE-71 behaves as a weak estrogen in both MCF-7 breast cancer cell proliferation and the ovariectomized adult mouse models. The anti-estrogenic activity may be due to an interaction with ERs, not to a metabolic depletion of coadministered estrogens. In animal studies, treatment route and duration determined whether DE-71 was estrogenic or not. BALB/c mice are more susceptible to DE-71 effects in estrogen target tissues and in liver than C57BL/6 mice. DE-71 also increased liver weight in both mouse strains tested, and this effect was not dependent on ER-α. It still remains to be seen if the above listed effects of DE-71 are due to the original BDE congeners or to their hydroxylated metabolites.
